# The Molecular Mechanisms of *Panax ginseng* in Treating Type 2 Diabetes Mellitus: Network Pharmacology Analysis and Molecular Docking Validation

**DOI:** 10.1155/2022/3082109

**Published:** 2022-09-16

**Authors:** Minh Nhat Tran, Sanghun Lee

**Affiliations:** ^1^Korean Medicine Data Division, Korea Institute of Oriental Medicine, Daejeon 34054, Republic of Korea; ^2^Korean Convergence Medical Science, University of Science and Technology, Daejeon 34113, Republic of Korea; ^3^Faculty of Traditional Medicine, Hue University of Medicine and Pharmacy, Hue University, Thua Thien Hue 49000, Vietnam

## Abstract

**Background:**

Type 2 diabetes mellitus (T2DM) is a chronic metabolic disorder with a high global incidence. *Panax ginseng* has been used to treat T2DM in traditional medicine, with previous *in vitro*, *in vivo*, and clinical trial studies demonstrating its efficacy. This study aimed to determine the mechanism of *P. ginseng* in treating T2DM by network pharmacology.

**Methods:**

The bioactive compounds of *P. ginseng* and corresponding targets of *P. ginseng-*T2DM were retrieved across multiple databases. The protein-protein interaction network was established using the STRING database and topological analysis helped identify the core target. Using the DAVID tool, we performed Gene Ontology (GO) and Kyoto Encyclopedia of Genes and Genomes (KEGG) enrichment analysis. Finally, we checked the binding of core targets and bioactive compounds using molecular docking.

**Results:**

The *P. ginseng*-T2DM networks mainly contained 22 bioactive compounds and 314 overlapping targets. The five most significant core targets were SRC, STAT3, MAPK1, AKT1, and PIK3R1. There were 244 GO terms and 95 KEGG pathways (adjusted *p* < 0.01) that were strongly correlated with diabetes-related signaling pathways, such as insulin resistance, the HIF-1 signaling pathway, the PI3K/Akt signaling pathway, the prolactin signaling pathway, the Rap1 signaling pathway, the Ras signaling pathway, the calcium signaling pathway, and the FoxO signaling pathway. Molecular docking results revealed that the top five core targets had a high binding affinity with the bioactive compounds of *P. ginseng*.

**Conclusion:**

The bioactive compounds and targets in *P. ginseng* ameliorate T2DM by regulating insulin resistance and multiple signaling pathways.

## 1. Introduction

Type 2 diabetes mellitus (T2DM) is a type of diabetes marked by elevated blood glucose levels resulting from defective insulin secretion and insulin resistance [1]. T2DM constitutes more than 90% of the 415 million diabetes cases found at present. It leads to cardiovascular and cerebrovascular diseases, blindness, and renal failure that endanger patients' mental and physical health and put a considerable strain on healthcare systems [1, 2]. Despite the availability of oral antidiabetic drugs, such as metformin, thiazolidinediones, meglitinides, and sulfonylureas [3], which have beneficial effects in patients with T2DM, these medications target specific chemicals that have been linked to their side effects [4]. Thus, traditional medicine is gaining attention as a safer and more cost-effective alternative medicine for T2DM [5].

*Panax ginseng* holds a prominent place in the oriental pharmacopeia. Traditionally, *P. ginseng* has long been used as an aphrodisiac, adaptogen, and nourishing stimulant as well as in the treatment of sexual dysfunction and T2DM [6]. Recently, clinical trials have indicated that *ginseng-*related therapies increase insulin sensitivity in patients with impaired glucose tolerance or T2DM [7]. Ginsenosides, the major compounds of *P. ginseng*, have been verified to have antidiabetic properties because of their antihyperglycemic, antiinflammatory, and antioxidant actions [8]. Current research on the mechanisms of *P. ginseng* compounds in the treatment of diabetes has concentrated on isolating compounds and studying their hypoglycemic effects *in vitro* and *in vivo* [9–11]. However, because of the multicompound and multitarget properties of traditional medicine, the underlying mechanisms of *P. ginseng* in T2DM treatment have not been comprehensively revealed.

Along with the advancement of bioinformatics, the integration of network pharmacology and molecular docking has been applied in recent years to study herbal compounds and traditional medicine mechanisms [12]. Network pharmacology is a new interdisciplinary field to understand the mechanisms of drugs within interconnected biological networks, so network pharmacology can aid in building “herb-multiple compounds-multiple targets” networks to discover the complex mechanisms of herbal medicines [13, 14]. Molecular docking is a computational approach for predicting the tentative binding and intermolecular interactions of bioactive compounds and targets derived from network pharmacology [15]. Hence, we combined network pharmacology with molecular docking to understand the antidiabetic mechanisms of *P. ginseng* systemically.

**Figure 1:**
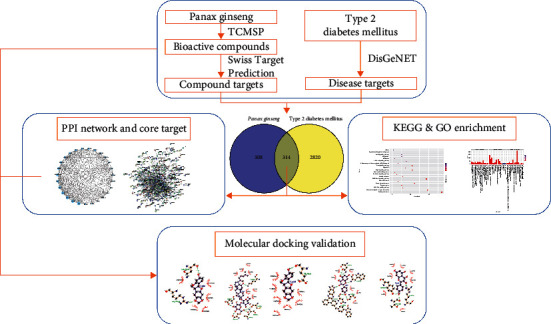
Workflow of the study.

In the current study, a systematic method was implemented to determine the therapeutic impact of *P. ginseng* on the effectiveness of T2DM treatment. Initially, we screened the bioactive compounds of *P. ginseng* and overlapping targets of *P. ginseng* and T2DM. Then, the protein-protein interaction (PPI) and core targets were established based on network topological structure analysis. Subsequently, we used Gene Ontology (GO) and Kyoto Encyclopedia of Genes and Genomes (KEGG) enrichment analysis to determine the functions and pathways of the overlapping targets. Lastly, we used molecular docking to check compound-target binding affinity based on network pharmacology results. The workflow is shown in Figure 1.

## 2. Materials and Methods

### 2.1. Bioactive Compounds Found in *P. ginseng* Screening

The compounds of *P. ginseng* were retrieved from the Traditional Chinese Medicine Systems Pharmacology Database and Analysis Platform (TCMSP) version 2.3 [16]. Based on the pharmacokinetic properties [17], these compounds were identified as bioactive compounds based on meeting the two criteria, i.e., drug-likeness (DL) ≥0.18 and oral bioavailability (OB) ≥30%. Furthermore, their corresponding names, PubChem compound IDs, and Chemical Abstracts Service (CAS) numbers were entered into the PubChem database (https://pubchem.ncbi.nlm.nih.gov/) to acquire the compounds' structures for target prediction and molecular docking.

### 2.2. Identification of Compound Targets for the Treatment of T2DM

The *Homo sapiens* targets associated with bioactive compounds were predicted using Swiss target prediction, in which we set the probability filter above zero [18]. Furthermore, the UniProt database (https://www.uniprot.org/) was used to identify the target names.

The disease targets of T2DM were obtained using DisGeNET version 7.0 with “diabetes mellitus, non-insulin-dependent” (CUI: C0011860) as the keywords, and all proposed disease targets have been selected [19]. The overlapping targets of *P. ginseng* compounds and T2DM were visualized using VENNY 2.1 [20].

### 2.3. Protein-Protein Interaction Network Construction

To evaluate the protein interactions among the overlapping targets, the PPI network was constructed using the Search Tool for the Retrieval of Interacting Genes/Proteins (STRING) database version 11.5 by setting the species as “*H. sapiens,*” and the highest confidence level as 0.9; the unconnected proteins were then removed [21]. Following this, the Cytoscape 3.9.0 software was used to construct a topology network in which the degree of a node was determined, which is defined as the number of connections that it has to other nodes [22]. The core targets were chosen based on nodes with degree values above twice the average.

### 2.4. Gene Ontology and Kyoto Encyclopedia of Genes and Genomes Pathway Analysis

To explore the detailed molecular mechanism of *P. ginseng*-related to the treatment of T2DM, we used the Database for Annotation, Visualization, and Integrated Discovery (DAVID) version 6.8 with an adjusted *p* value <0.01 (after Benjamini's correction) to process GO and KEGG pathway enrichment [23]. GO analyses primarily focus on the target's biological processes, molecular functions, and cellular components, while KEGG pathway enrichment analyses focus on the target's multiple pathways and activities. We constructed an “herb-compound-target-pathway” network diagram using Cytoscape 3.9.0 to explore the mechanisms of *P. ginseng* [22].

### 2.5. Molecular Docking

Molecular docking was used to calculate docking energy between the top five core targets and their corresponding bioactive compounds. The 3D structures of ligands (compounds) and protein receptors (targets) were downloaded from the PubChem database (https://pubchem.ncbi.nlm.nih.gov/) and Protein Data Bank (https://www.rcsb.org/), respectively. The PyMOL 2.5.2 software was used to create the chemical structures [24]. Then, each pair of the compound and target was imported into AutoDock Tools 1.5.7 to add polar hydrogen, remove water molecules, and obtain the grid box for molecular docking. The Autodock Vina 1.1.2 was used to calculate the docking energy [25]. Commonly, the target possesses a considerable binding capacity with the compound if the docking energy between receptor and ligand is less than −5 kcal/mol. The lowest binding energy of the ligand-protein interaction of each corresponding target was chosen to visualize the interactions using Ligplot+ 2.2.4 software [26].

## 3. Results

### 3.1. Bioactive Compounds of *P. ginseng*

After retrieving the compounds from the TCMSP database, 190 related compounds of *P. ginseng* were obtained. Altogether, 22 compounds were screened as bioactive compounds of *P. ginseng* (Table 1) with OB ≥ 30% and DL ≥ 0.18.

### 3.2. *P. ginseng* and T2DM Overlapping Targets

A total of 622 targets for 20 bioactive compounds of *P. ginseng* were predicted by the SwissTargetPrediction database. There were no targets for chrysanthemaxanthin and malkangunin.

**Figure 2:**
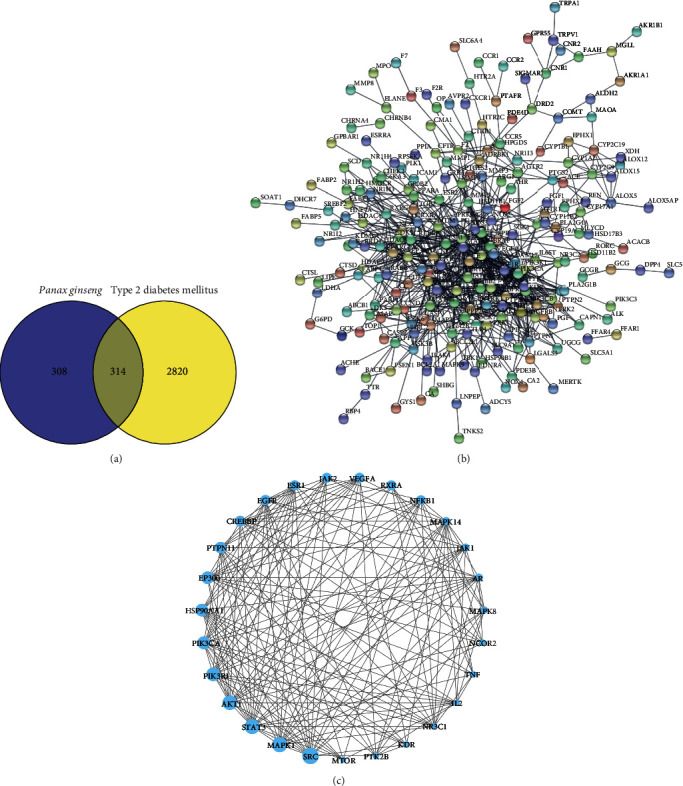
The overlapping targets and their protein-protein interaction networks. (a) Overlapping targets of *Panax ginseng* and type 2 diabetes mellitus. (b) Protein-protein interaction network of overlapping targets. (c) Protein-protein interaction network of 27 core targets. The circular nodes represent targets and are ordered by node size: the larger the node size, the more important the target.

By screening the DisGeNET database, 3134 T2DM-related targets were obtained. The 622 compound targets were mapped to the 3134 T2DM targets, and 314 overlapping targets were available, as shown in Figure 2(a).

### 3.3. Protein-Protein Interaction Network

In order to analyze the 314 overlapping targets, they were input into the STRING database to construct the PPI network (Figure 2(b)). The network was then imported into Cytoscape 3.9.0, which resulted in a network model with 235 nodes and 895 edges. According to topological analyses, 27 core targets were selected with a node degree value larger than the two-fold average, including SRC, STAT3, MAPK1, AKT1, PIK3R1, PIK3CA, HSP90AA1, EP300, PTPN11, CREBBP, EGFR, JAK2, ESR1, RXRA, VEGFA, NFKB1, MAPK14, AR, JAK1, MAPK8, NCOR2, TNF, IL2, NR3C1, MTOR, PTK2B, and KDR. As shown in Figure 2(c), a network diagram of the 27 core targets was constructed. Among them, SCR had the highest degree value (degree = 47), followed by STAT3 (degree = 43), MAPK1 (degree = 43), AKT1 (degree = 41), and PIK3R1 (degree = 39). As a result, these five targets were considered to be the central genes of *P. ginseng* in T2DM treatment and were selected for molecular docking.

### 3.4. Gene Ontology and Kyoto Encyclopedia of Genes and Genomes Pathway Enrichment Analysis

**Figure 3:**
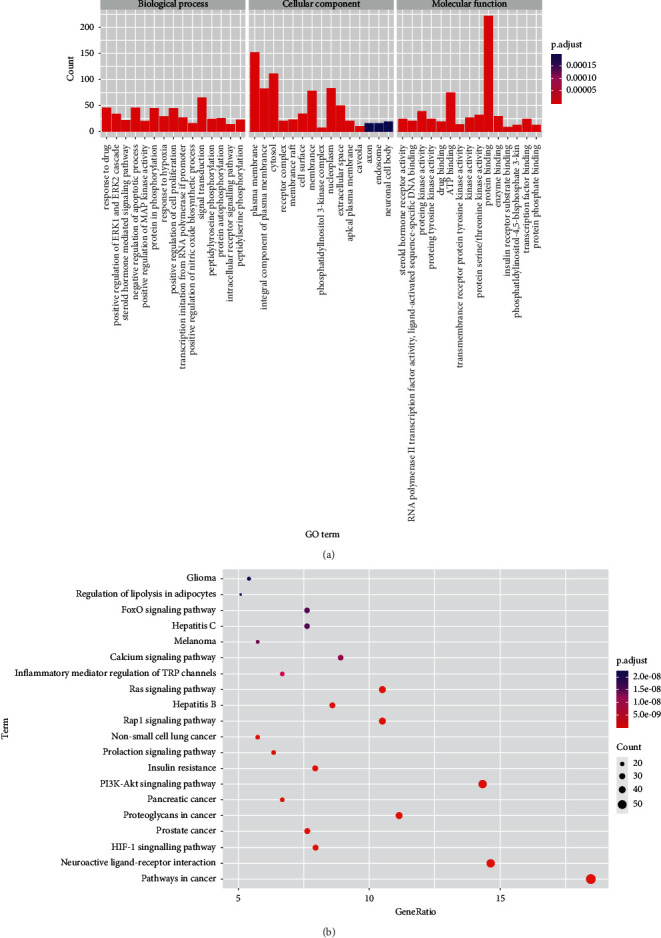
KEGG and GO pathway enrichment analysis of overlapping targets (a) The top 15 GO enrichment terms of three categories. (b) The top 20 KEGG pathway enrichment terms.

The GO enrichment analysis of 314 targets resulted in 244 GO terms (adjusted *p* < 0.01), including 173 biological process terms, 44 molecular functions, and 27 cell compositions. The top five terms of biological process, ranked through the adjusted *p* value were as follows: response to the drug (GO: 0042493), positive regulation of the ERK1 and ERK2 cascade (GO: 0070374), steroid hormone-mediated signaling pathway (GO: 0043401), negative regulation of the apoptotic process (GO: 0043066), and positive regulation of MAP kinase activity (GO: 0043406). The top five terms in molecular functions were as follows: steroid hormone receptor activity (GO: 0003707), RNA polymerase II transcription factor activity, ligand-activated sequence-specific DNA binding (GO: 0004879), protein kinase activity (GO: 0004672), protein tyrosine kinase activity (GO: 0004713), and drug binding (GO: 0008144). Moreover, these processes occurred mainly in the plasma membrane (GO: 0005886), integral compounds of the plasma membrane (GO: 0005887), cytosol (GO: 0005829), receptor complex (GO: 0043235), and membrane raft (GO: 0045121). The top 15 GO terms of the three categories are visualized in Figure 3(a).

**Figure 4:**
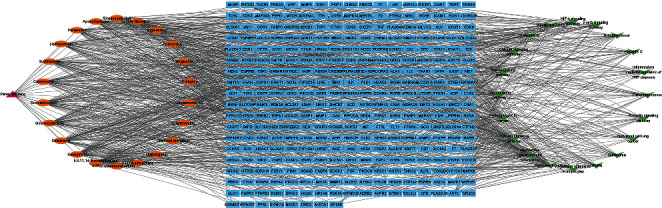
Network herb-compound-target-pathway of *Panax ginseng* in type 2 diabetes mellitus treatment. The pink triangle indicates the herb, the orange ellipse indicates the compound, the blue square indicates the target, and the green V-shape indicates the pathway.

**Figure 5:**
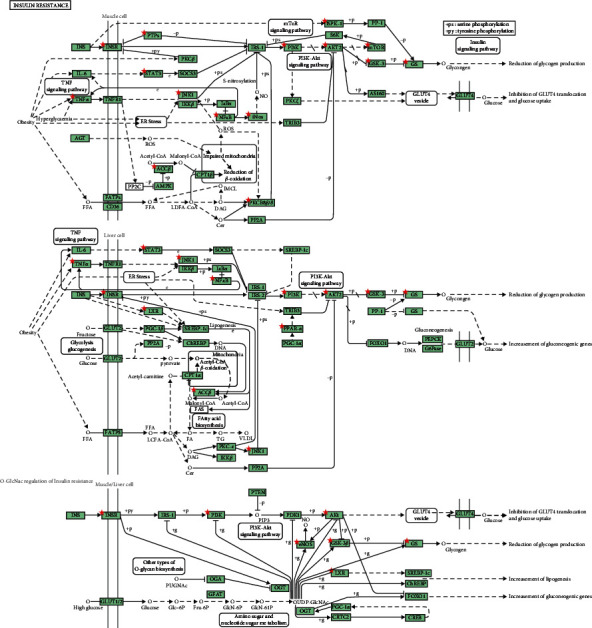
Relevant targets (red stars) of *Panax ginseng* in the insulin resistance pathway.

KEGG pathway enrichment analysis obtained 95 signaling pathways (adjusted *p* < 0.01), mainly involved pathways in cancer (hsa05200), neuroactive ligand-receptor interaction (hsa04080), the PI3K/Akt signaling pathway (hsa04151), proteoglycans in cancer (hsa05205), the Rap1 signaling pathway (hsa04015), and the Ras signaling pathway (hsa04014). The top 20 pathways are visualized in Figure 3(b). The information on herb, 22 bioactive compounds, 314 overlapping targets, and top 20 pathways was imported into Cytoscape to create the “herb-compound-target-pathway” network (Figure 4). Relevant targets of *P. ginseng* in the insulin resistance pathway are shown in Figure 5.

### 3.5. Molecular Docking Results

Based on the PPI network, the top five core targets (SCR, STAT3, MAPK1, AKT1, and PIK3R1) and the corresponding bioactive compounds of *P. ginseng* were selected for molecular docking. The docking energy scores are shown in Table 2, the lower the docking energy, the better the binding capacity. Particularly, (+)-maackiain had the lowest docking energy scores to SRC and MAPK1; kaempferol had the lowest docking energy scores to AKT1 and PIK3R, whereas ginsenoside Rg5 had the lowest docking energy to STAT3. All binding docking energy scores were lower than −5 kcal/mol, implicating that the predicted core targets combined stably with *P. ginseng*.

## 4. Discussion

In traditional medicine, diabetes is classified as “Xiaoke.” The primary pathogenesis of “Xiaoke” is spleen deficiency, the root pathogen is a lack of Qi spirit, and blood stasis is implicated during the entire course of diabetes. In clinical treatment, *P. ginseng,* a Qi-tonifying herb, is used in combination with other T2DM treating herbs, such as “white tiger plus ginseng,” to invigorate the Qi [27, 28]. The antihyperglycemic effect of *P. ginseng* has also been demonstrated by previous *in vitro*, *in vivo*, and clinical trial studies [6]. Using a systematic combination of network pharmacology and molecular docking in this study, we found the bioactive compounds and related targets of *P. ginseng* against T2DM in multiple signaling pathways.

According to the screening results, the treatment activity of *P. ginseng* was discovered to be associated with 22 bioactive compounds (Table 1). Based on the “herb-compound-target-pathway” network, the degree values were the highest among the top four compounds: 5, 8, 11, 14-eicosatetraenoic acid, kaempferol, girinimbine, and ginsenoside Rh4 (Figure 4); therefore, these compounds were identified as the major compounds of *P. ginseng* against T2DM. 5, 8, 11,14-eicosatetraenoic acid, also calls arachidonic acid, can prevent type 1 and 2 diabetes. Arachidonic acid and its metabolites have been shown to regulate free radical generation, cell membrane fluidity, membrane receptors, voltage-gated ion channels, inflammation, nitric oxide formation, and immune responses, all of which impact the pathogenesis of diabetes [29]. Experimental animal models showed that arachidonic acid can significantly prevent whole-body insulin resistance [30], and increases LXA4 formation, which contributes to its antidiabetic and anti-inflammatory properties [31]. Kaempferol has been known to promote insulin sensitivity and preserve pancreatic *β*-cell mass. It also exhibits an antidiabetic effect via the inhibition of gluconeogenesis in the liver by reducing glucose-6 phosphatase and pyruvate carboxylase activity as well as increasing the activity of AKT and hexokinase [32]. Girinimbine has been shown to modulate apoptosis [33] and oxidative stress [34], two essential agents in the pathophysiology of T2DM. An experimental study identified the expression of proteins related to insulin/IGF-1 signaling on human cells treated with girinimbine [33]. Ginsenoside Rh4, ginsenoside rh2, panaxadiol, and ginsenoside Rg5 are the main compounds in *P. ginseng* and are called ginsenosides. They improve insulin resistance by reducing hepatic glucose production and lipid accumulation through the activation of GSK3*β* and by suppressing both FOXO1 and SREBP transcription [8]. In addition, stigmasterol and *β*-sitosterol have demonstrated potential antidiabetic effects through *in vitro* and *in vivo* studies [35–37].

Based on the multicompound and multitarget features of network pharmacology, 314 overlapping targets were found between the herb and disease. As seen in the PPI topological network (Figures 2(b) and 2(c)), 27 core targets were identified; among them, SRC, STAT3, MAPK1, AKT1, and PIK3R1 were the top five core targets that play an essential role in T2DM treatment by *P. ginseng*. In an animal study, SRC activation led to collagen accumulation and the mitogen-activated protein kinase (MAPK) signaling pathway, suggesting that it might be a therapeutic target for diabetic nephropathy [38]. STAT3 is associated with IL-6-induced insulin resistance in human skeletal muscle [39]. STAT3 has also been reported to sensitize insulin signaling by negatively regulating GSK-3*β*, a negative regulator of insulin [40]. MAPK1 is known to suppress STAT3 activation enzymatically and is proposed as a valuable candidate for diabetes therapy [41]. MAPK1 phosphorylates and negatively modulates STAT3, which is essential for normal glucose homeostasis [42,43]. AKT1 is one of three AKT kinases that can control glucose absorption into fat cells and muscles by increasing GLUT4 glucose transporter translocation. By suppressing the expression of glucose 6-phosphatase and phosphoenolpyruvate carboxykinase, Akt also represses liver gluconeogenesis [44]. PIK3R1 encodes the subunits of class Ia phosphoinositide 3-kinase (PI3K), which is key for insulin signaling [45, 46]. PIK3R1 mutations in humans result in severe insulin resistance and PI3K-dependent signaling [46]. At the same time, a lack of PIK3R1 in mice resulted in increased glucose tolerance and insulin sensitivity [47]. In addition, the top five core targets (SRC, STAT3, MAPK1, AKT1, and PIK3R1) were successfully checked by molecular docking. These core targets are stably bound to the respective bioactive compounds. In summary, the above results show that core targets of *P. ginseng* have significant antidiabetic effects.

In the KEGG enrichment analysis, insulin resistance and many diabetes-related signaling pathways were identified, such as the HIF-1, PI3K/Akt, prolactin, Rap1, Ras, calcium, and FoxO signaling pathways (Figure 3(b)). The insulin resistance pathway is common in people with T2DM, obesity, cardiovascular disease, or nonalcoholic fatty liver disease. In Figure 5, it is shown that relevant targets participated in multipathway of insulin resistance, such as enhanced phosphorylation of the insulin receptor substrate protein, enhanced IRS-1 proteasome degradation, reduced activation of Akt and PI3K, and increased phosphatase activity. As a result of insulin resistance, glucose uptake, glycogen synthesis in skeletal muscle, and glycogen synthesis in the liver decrease, while hepatic gluconeogenesis increases [48]. HIF-1 is the transcription factor that enhances hypoxia adaption and relates to T2DM complications such as diabetic retinopathy [49] and diabetic foot ulcers [50]. The PI3K/Akt pathway is a multifunctional signaling pathway that is critical in the pathophysiology of diabetic nephropathy and is activated in high glucose-stimulated HKC cells [51]. Ca^2+^ signaling and its dysregulation have been linked to diabetes development, and it plays a vital role in Cd-induced *β*-cell dysfunction and apoptosis [52]. Ca^2+^/cAMP signaling regulates insulin release from pancreatic *β*-cells and contribute to *β*-cells homeostasis by stimulating cell proliferation and differentiation [53]. In addition, the signaling pathways of prolactin, Rap1, Ras, and FoxO are involved in insulin production or the pathology of diabetes [54–57]. In summary, the mechanism of the antidiabetic treatment involves *P. ginseng* acting through multitarget and multipathway.

The combination of network pharmacology and molecular docking can provide compelling evidence of the molecular mechanisms of herbal medicine. However, there are also several limitations to our study. First, more comprehensive input data are required to make the analysis outcomes more reliable. Second, the computational tools of network pharmacology and molecular docking for predicting and validating the mechanisms of herbal medicine still have drawbacks. For example, SwissTargetPrediction uses ligand-based approaches to target prediction based on the assumption that if the molecule is active, which means the molecule will likely bind to some protein. For molecules with unknown bioactivity, predicted targets are false positives [58]. Therefore, our results need further scientific verification.

## 5. Conclusions

This study revealed the “multicompound, multitarget, and multipathway” mechanisms of *P. ginseng* for treating T2DM by using network pharmacology. The outcomes showed that 22 bioactive compounds and 314 corresponding targets significantly contributed to the antidiabetic effects of *P. ginseng* via regulating diabetes-related signaling pathways and biological processes, such as insulin resistance, the HIF-1 signaling pathway, the PI3K/Akt signaling pathway, the prolactin signaling pathway, the Rap1 signaling pathway, the Ras signaling pathway, the calcium signaling pathway, and the FoxO signaling pathway. Moreover, molecular docking successfully checked the binding action of the top five core targets and their corresponding bioactive compounds.

## Figures and Tables

**Table 1:** Twenty-two bioactive compounds of *Panax ginseng.*

Molecular name	PubChem CID	TCMSP ID	Oral bioavailability (%)	Drug-likeness
(+)-Maackiain	161298	MOL003648	65.83	0.54
5, 8, 11, 14-Eicosatetraenoic acid	5312542	MOL005320	45.57	0.2
Aponorhyoscine	5319581	MOL005308	66.65	0.22
Beta-sitosterol	222284	MOL000358	36.91	0.75
Celabenzine	442847	MOL005314	101.88	0.49
Chrysanthemaxanthin	21160900	MOL004492	38.72	0.58
Deoxyharringtonine	285342	MOL005317	39.27	0.81
Dianthramine	441562	MOL005318	40.45	0.2
Diisooctyl phthalate	33934	MOL002879	43.59	0.39
Frutinone A	441965	MOL005321	65.9	0.34
Ginsenoside Rg5	11550001	MOL005401	39.56	0.79
Ginsenoside Rh2	119307	MOL005344	36.32	0.56
Ginsenoside Rh4	21599928	MOL005348	31.11	0.78
Girinimbine	96943	MOL005356	61.22	0.31
Kaempferol	5280863	MOL000422	41.88	0.24
Malkangunin	90473155	MOL005360	57.71	0.63
Panaxadiol	73498	MOL005376	33.09	0.79
Protopine	4970	MOL000787	59.26	0.83
Schisantherin B	6438572	MOL005357	31.99	0.83
Sitogluside	5742590	MOL005399	36.91	0.75
Stigmasterol	5280794	MOL000449	43.83	0.76
Suchilactone	132350840	MOL005384	57.52	0.56

**Table 2:** Docking scores of SCR, STAT3, MAPK1, AKT1, and PIK3R1 with corresponding bioactive compounds.

Protein	PDB ID	Compounds	Affinity (kcal/mol)	Best-docked complex
SRC	1FMK	(+)-Maackiain	−8.6	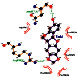
		Kaempferol	−8.0	
		Aponorhyoscine	−7.4	

STAT3	6NJS	Ginsenoside Rg5	−8.1	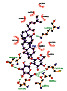
		Ginsenoside Rh4	−7.8	
		Sitogluside	−7.7	
		Ginsenoside rh2	−7.7	

MAPK1	4G6O	(+)-Maackiain	−8.3	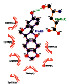
		Ginsenoside Rh4	−8.0	
		Panaxadiol	−7.3	
		Schisantherin B	−6.1	

AKT1	3O96	Kaempferol	−8.5	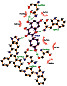
		Girinimbine	−8.3	
		Aponorhyoscine	−6.8	

PIK3R1	3I5S	Kaempferol	−7.1	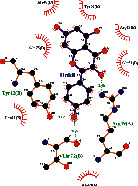
		Frutinone A	−6.8	
		Suchilactone	−6.5	
		Ginsenoside Rh4	−10.3	
		Protopine	−10.0	
		Celabenzine	−9.1	
		Schisantherin B	−8.2	
		(+)-Maackiain	−8.4	
		Suchilactone	−7.9	

## Data Availability

The data used to support the findings of this study are available from the corresponding author upon request.
